# Theoretical Study of the Photolysis Mechanisms of Methylpentaphenyldimetallanes (Ph_3_MM′Ph_2_Me; M, M′ = Si and Ge)

**DOI:** 10.3390/molecules23061351

**Published:** 2018-06-04

**Authors:** Shih-Hao Su, Ming-Der Su

**Affiliations:** 1 Department of Applied Chemistry, National Chiayi University, Chiayi 60004, Taiwan;su83129@yahoo.com.tw; 2 Department of Medicinal and Applied Chemistry, Kaohsiung Medical University, Kaohsiung 80708, Taiwan

**Keywords:** photolysis, aryldisilanes, triplet states, intersystem crossing, spin crossover

## Abstract

The mechanisms of the photolysis reactions are studied theoretically at the M06-2X/6-311G(d) level of theory, using the four types of group 14 molecules that have the general structure, Ph_3_M–M′Ph_2_Me (M and M′ = Si and Ge), as model systems. This study provides the first theoretical evidence for the mechanisms of these photorearrangements of compounds that contain a M–M′ single bond. The model investigations indicate that the preferred reaction route for the photolysis reactions is, as follows: reactant → Franck-Condon (FC) region → minimum (triplet) → transition state (triplet) → triplet/singlet intersystem crossing → photoproducts (both di-radicals and singlets). The theoretical findings demonstrate that the formation of radicals results from reactions of the triplet states of these reactants. This could be because both the atomic radius and the chemical properties of silicon and germanium are quite similar to each other and compared to other group 14 elements, their photolytic mechanisms are nearly the same. The results for the photolytic mechanisms that are studied in this work are consistent with the available experimental observations and allow for a number of predictions for other group 14 dimetallane analogues to be made.

The photochemistry of disilanes that have various substituents have attracted intense interest because they are used in the study of fundamental chemistry problems, as well as for their potential applications [1,2,3,4,5,6,7,8,9,10,11,12,13,14,15,16,17,18,19,20,21,22,23]. It is established that the direct irradiation of aryldisilanes in solution results in the formation of transient silenes, which are formally derived from the disproportionation and recombination of the silyl free radicals that are developed in the homolysis of the Si–Si single bond (Scheme 1) [24,25,26,27,28,29,30,31,32,33,34,35,36,37,38,39,40,41]. Although there has been much experimental study of the photoreactions of aryldisilanes and their derivatives, to the authors’ best knowledge, no theoretical studies deal with the photochemical mechanisms of these silicon-based compounds.

These interesting experimental results for photochemical aryldisilane stimulate this study of the potential energy surfaces for these reactions, using density functional theory (DFT). A study of the photolysis reaction, Scheme 2, is thus detailed.

Scheme 2 is chosen as the model because there have been many experimental studies of methylpentaphenyldisilane photochemistry [24,25,26,27,28,29,30,31,32,33,34,35,36,37,38,39,40,41]. However, there have been no reported theoretical studies of these experimental results. In particular, there are no systematic theoretical computations that relate to the effect of the elements on the 1-methyl-1,1,2,2,2-pentaphenyldimetallane (**1**; Ph_3_M–M′Ph_2_Me; M and M′ = Si and Ge) systems. 

In order to give a realistic representation of the photochemical reaction of Ph_3_M–M′Ph_2_Me, all of the DFT computations are accomplished using the GAUSSIAN 09 package of programs [42] at the M06-2X/6-311G(d) [43] level of theory. All of the optimized structures were confirmed by normal mode vibrational analysis. Time-dependent density functional theory (TD-DFT) computations [44,45] were also performed with the same DFT and 6-311G(d) basis sets. The minimum energy of the intersystem crossing points between the singlet and triplet potential energy surfaces are also calculated using the GAUSSIAN 09 package, together with the code that was developed by Harvey et al. [46,47].

According to the model (Scheme 2) that was studied in this work, the lowest singlet (S_0_) or triplet (T_1_) excitation for the Ph_3_Si–SiPh_2_Me (**1-Si-Si**) is the σ^Si–Si^ (HOMO) → σ^Si–Si^* (LUMO) transition. It was experimentally reported that Ph_3_Si–SiPh_2_Me (**1-Si-Si**) was directly irradiated using 254-nm (=113 kcal/mol) lamps [24,25,26,27,28,29,30,31,32,33,34,35,36,37,38,39,40,41], which is in good agreement with the present computational data (117 kcal/mol). This inspires confidence that these model computations are trustworthy for these studies of the mechanisms of the photolysis reactions of Ph_3_Si–GePh_2_Me (**1-Si-Ge**), Ph_3_Ge–SiPh_2_Me (**1-Ge-Si**) and Ph_3_Ge–GePh_2_Me (**1-Ge-Ge**).

The TD-DFT computations [44,45] also demonstrate that the excited states (kcal/mol) increase in the order (Figure 1):

(a) For 1-Si-Si: [1-Si-Si(T_1_)]* (95.2) < [1-Si-Si(T_2_)]* (106.3) < [1-Si-Si(S_1_)]* (108.6) < [1-Si-Si(T_3_)]* (117.2) < [1-Si-Si(S_2_)]* (120.6).
(b) For 1-Si-Ge: [1-Si-Ge(T_1_)]* (99.7) < [1-Si-Ge(T_2_)]* (104.8) < [1-Si-Ge(S_1_)]* (107.3) < [1-Si-Ge(T_3_)]* (114.6) < [1-Si-Ge(S_2_)]* (121.9).
(c) For 1-Ge-Si: [1-Ge-Si(T_1_)]* (100.8) < [1-Ge-Si(T_2_)]* (105.2) < [1-Ge-Si(S_1_)]* (110.6) < [1-Ge-Si(T_3_)]* (116.2) < [1-Ge-Si(S_2_)]* (120.0).
(d) For 1-Ge-Ge: [1-Ge-Ge(T_1_)]* (98.4) < [1-Ge-Ge(S_1_)]* (108.3) < [1-Ge-Ge(T_2_)]* (117.2) < [1-Ge-Ge(T_3_)]* (123.5) < [1-Ge-Ge(S_2_)]* (124.4).

Namely, the theoretical evidences indicate that the lowest-lying excited state for these four molecules (**1-Si-Si**, **1-Si-Ge**, **1-Ge-Si**, and **1-Ge-Ge**) is the first excited triplet state (T_1_). It is well known that spin-allowed absorption cross-sections are typically larger than those for spin-forbidden excitations. As a result, photo-excitation promotes these four molecules to a singlet excited electronic state (S_1_), and the molecule subsequently relaxes, branching between the T_1_ and S_0_ states, so considerations start on the triplet state energy surface. This computational data strongly suggest that the photolysis reactions of **1** must firstly progress on the triplet states and must only include the σ → σ* transition. As a result, the triplet state surfaces are the main focus of this study.

The essential characteristic of the photochemical mechanisms of **1** is the location of the intersystem crossing in the excited-triplet and the ground-singlet electronic states. In this study, since triphenylsilyl and methyldiphenylsilyl radicals are observed as photoproducts of **1-Si-Si** (Scheme 1) [24,25,26,27,28,29,30,31,32,33,34,35,36,37,38,39,40,41], the Si–Si single bond in **1-Si-Si** must be broken. Accordingly, a Si–Si σ bond cleavage mechanism is suggested for the conversion of **1-Si-Si** to triphenylsilyl and methyldiphenylsilyl radicals, and this is shown in Figure 2(i) [48]. It is worthy of note that although the silicon-silicon σ bond cleavage does not require full optimization of **1-Si-Si**, this at least shows that there is degeneracy between a HOMO (σ) and a LUMO (σ*) when the silicon-silicon single bond is lengthened. Increasing the M–M′ single bonds in **1-Si-Ge**, **1-Ge-Si**, and **1-Ge-Ge**, can also lead to degeneracy of the S_0_ and T_1_ energy surfaces at about 4.0 Å, which are estimates, as shown in Figure 2(ii–iv), respectively. These results are used to explain the mechanisms for the photolysis reactions of **1-Si-Si**, **1-Si-Ge**, **1-Ge-Si** and **1-Ge-Ge** in the following study.

In order to understand the similarities and differences between the four types of photolysis reactions (Figure 2), the reaction profiles are shown in Figure 1, which also contains the relative energies for all of the critical points with respect to the energy of the corresponding reactants **1**. The optimized geometrical structures are all collected in Figure 3 (Ph_3_Si–SiPh_2_Me), Figure 4 (Ph_3_Si–GePh_2_Me), Figure 5 (Ph_3_Ge–SiPh_2_Me), and Figure 6 (Ph_3_Ge–GePh_2_Me), respectively. 

The potential energy surfaces for the photolytic mechanisms (Scheme 2) that are studied in this work are analogous to each other. That is to say, these theoretical computations suggest that the reaction mechanisms for all of the photolysis reactions for **1** proceed, as follows:

1(S_0_) + hν → (1-T_1_)* → 1-T_1_ − Min → 1-T_1_-TS → 1-T_1_/S_0_ → 2,3-S_0_ → 4,5-S_0_ and **6-S_0_**.

In other words, the reactant (**1**) is initially excited to its lowest-lying triplet state (T_1_), which is denoted as (**1-T_1_**)*, by absorbing light. From this (**1-T_1_**)* point, the molecule relaxes to a local minimum (**1-T_1_-Min**) near to the S_0_ geometry. When the M–M′ bond distance is increased, the transition state (**1**-**T_1_**-**TS**) is obtained, which is still located on the triplet state surface. The experimental observations [24,25,26,27,28,29,30,31,32,33,34,35,36,37,38,39,40,41] show that the photolysis reaction for **1-Si-Si** is non-adiabatic [49]. The reaction begins from the triplet surface and advances ultimately along the ground singlet state pathway. Therefore, the intersystem crossing point of the T_1_ and S_0_ surfaces must play a key role in the mechanistic photolysis reactions of **1**. As illustrated in Figure 1, the spin crossover from the triplet state (T_1_) to the ground singlet state (S_0_) states occurs in the region of the T_1_/S_0_ intersection (**1-T_1_/S_0_**). The results given in Figure 1 show that relaxing through **1-T_1_/S_0_** may create the first photoproducts that have doublet radicals (i.e., **2,3-S_0_**; Ph_3_M· and ·M′Ph_2_Me; path (I)). When the dispropotionation (path (II)) and the recombination (path (III)) processes are complete, the other singlet photoproducts are produced. These are **4,5-S_0_** (Ph_3_M–H and H_2_C=M′Ph_2_) and **6-S_0_**.

The computational results outlined in Figure 1 allow for the following conclusions to be drawn:

(1) The theoretical studies using the M06-2X/6-311G(d) level of theory show that after the direct irradiation of aryldisilanes, arylgermasilanes, and aryldigermanes of the general structure, Ph_3_M–M′Ph_2_Me (**1**), they occupy the lowest excited triplet state and then proceed to M–M′ bond homolysis via the intersystem crossing, to acquire two doublet radicals, **2** and **3** (overall singlet). After the subsequent dispropotionation and recombination procedures, they finally generate a mixture of singlet photoproducts (**4**–**6**). It should be emphasized that this is the first theoretical verification that radicals are formed as a result of reactions of the triplet states of Ph_3_M–M′Ph_2_Me (**1**).
(2) Figure 1 clearly shows that the reactants **1** have an excess of energy, because of the vertical excitation to the Frank-Condon point at the triplet state (**1**-**T_1_**)*, which is larger than the subsequent activation barriers. That is to say, when Ph_3_M–M′Ph_2_Me (**1**) absorbs light, this molecule has sufficient internal energy to overcome the energy barriers to produce various types of photoproducts (**2–6**) without any difficulty. This theoretical finding for the **1-Si-Si** molecule is in good agreement with the experimental evidence [24,25,26,27,28,29,30,31,32,33,34,35,36,37,38,39,40,41]. Since there are no pertinent experimental or theoretical reports on the photolysis reactions of the compounds, **1-Si-Ge**, **1-Ge-Si,** and **1-Ge-Ge**, this result is a prediction.
(3) The theoretical observations that are shown in Figure 1 indicate that the relative energies of two monoradical species (Ph_3_M·and·M′Ph_2_Me) are higher than those of the other singlet photoproducts (**4–6**). The quantum yields of the former are estimated to be lower than those of the latter. This prediction is again consistent with the available experimental observations for the **1-Si-Si** molecule, in which it is reported that the formation of the corresponding silyl radicals (**2** and **3**), 1,1-diphenylsilene (**5**), and the silatriene (**6**) give respective yields of ca. 20%, 35%, and 42% [30]. Therefore, in the photolysis reactions for compounds **1-Si-Ge**, **1-Ge-Si** and **1-Ge-Ge**, the quantum yields for their singlet photoproducts **4**–**6** should be larger than those of the other two doublet radicals, **2** and **3 [50]**. 
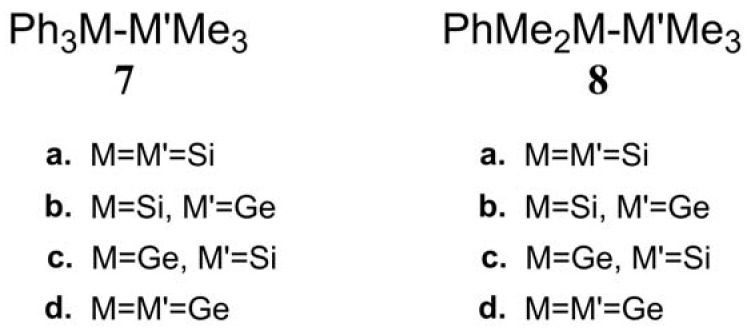
(4) The theoretical conclusions shown in Figure 1 also apply to other group 14 dimetallane analogues, such as 1,1,1-trimethyl-2,2,2-triphenyldimetallanes (**7**; Ph_3_M–M′Me_3_; M and M′ = Si and Ge) and 1,1,1,2,2-pentamethyl-2-phenyldimetallanes (**8**; PhMe_2_M–M′Me_3_; M and M′ = Si and Ge). That is to say, the direct irradiation of these compounds produces the triplet species, which then mainly results in M–M′ bond homolysis to generate the formation of a radical and other singlet photoproducts (similar to **4**–**6**), via radical disproportionation and recombination. Indeed, some earlier experimental studies of **7d** by Mochida and Hayashi [51] and a related those for series of molecules (**8b**–**8d**), as reported by Mochida and Gaspar [52,53] and their co-workers, show similar results that are in reasonable agreement with these theoretical predictions.

In conclusion, this M06-2X/6-311G(d) study presents the first theoretical explanation for the photolytic mechanisms of group 14 dimetallanes that contain a M–M′ single bond. This work demonstrates that the spin crossover plays a central role in the photolysis reactions of these compounds, from the excited triplet state to singlet ground state. In principle, the theoretical evidence that is given by this study demonstrates that both the potential energy surfaces and the order of the photochemical reactivity are similar for these group 14 dimetallanes, regardless of whether silicon or germanium elements are concerned. This may be because silicon and germanium are quite analogous to each other, in terms of both their atomic radius (117 pm and 122 pm for silicon and germanium, respectively) and their chemical properties [54,55]. Therefore, their photolytic behavior should be analogous, as outlined in Figure 1.

It is hoped that this work will stimulate further study of the photolysis reactions of substituted group 14 dimetallanes that have a M–M′ σ bond.

## Figures and Tables

**Scheme 1:**
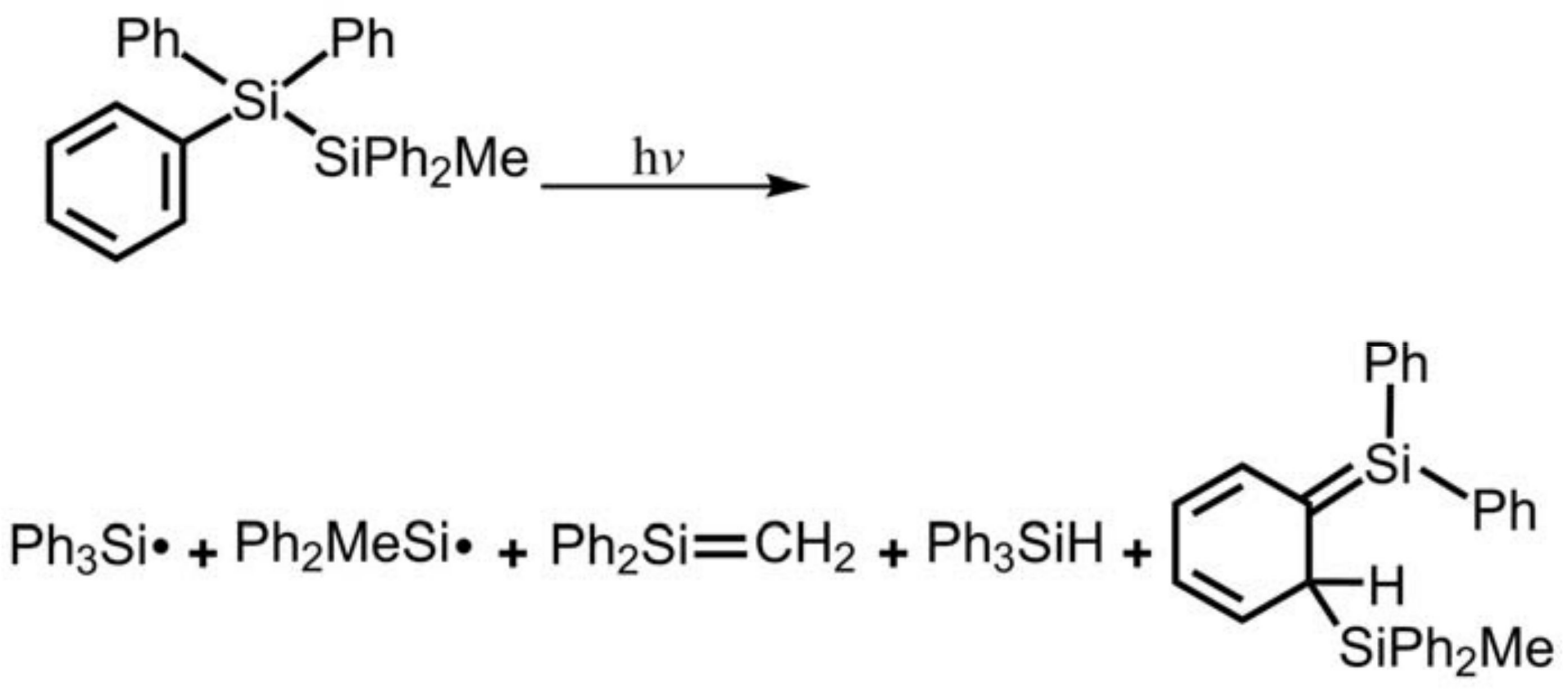
The photochemical reactions of aryldisilane.

**Scheme 2:**
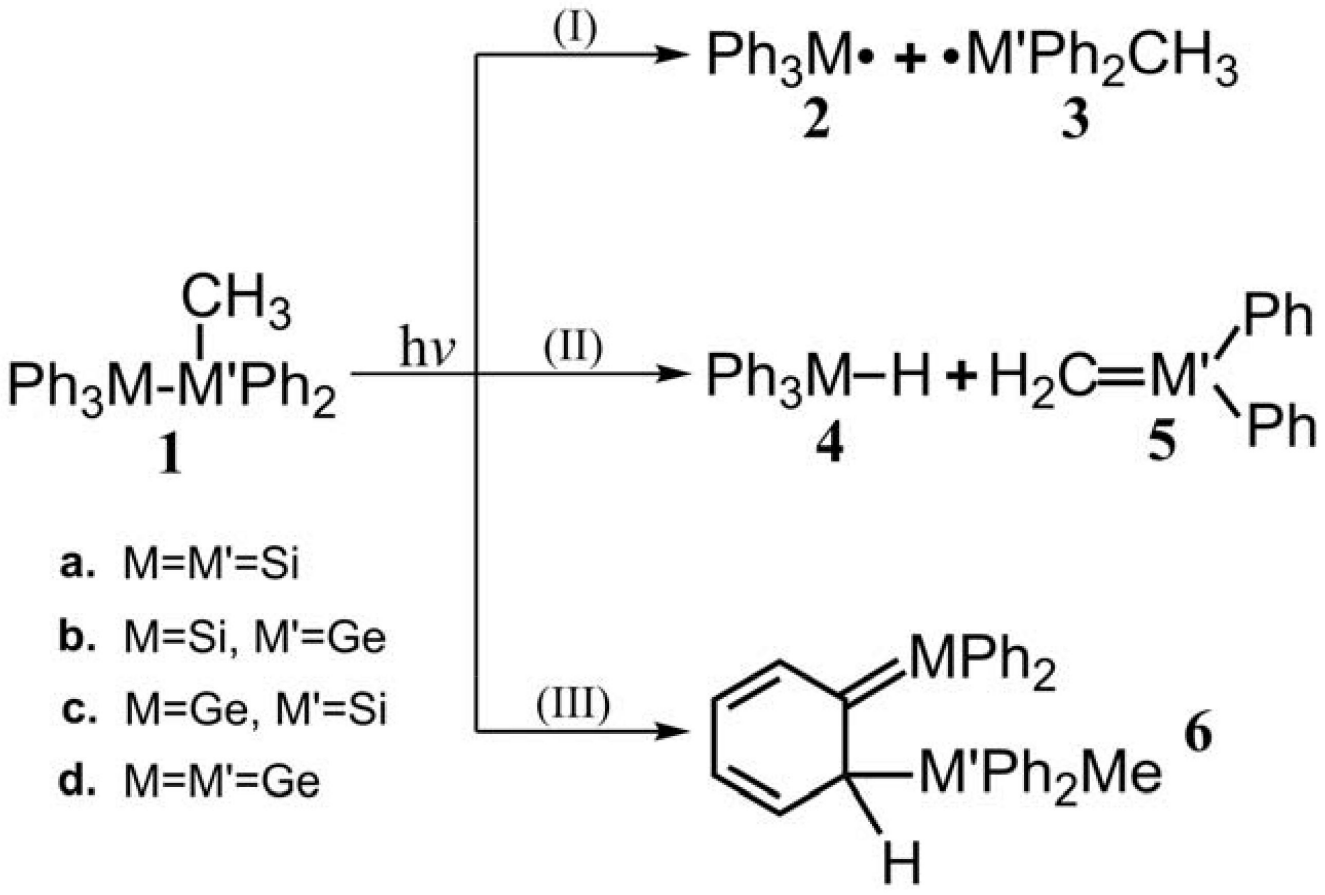
The experimental results. See the text.

**Figure 1:**
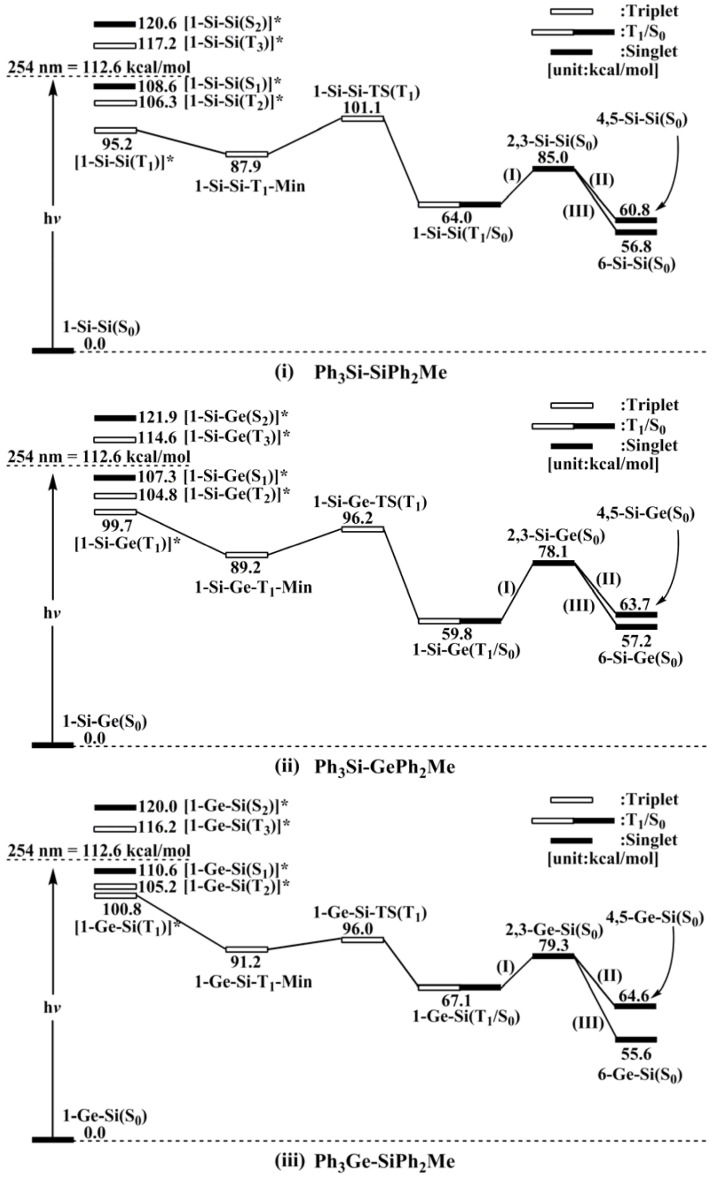

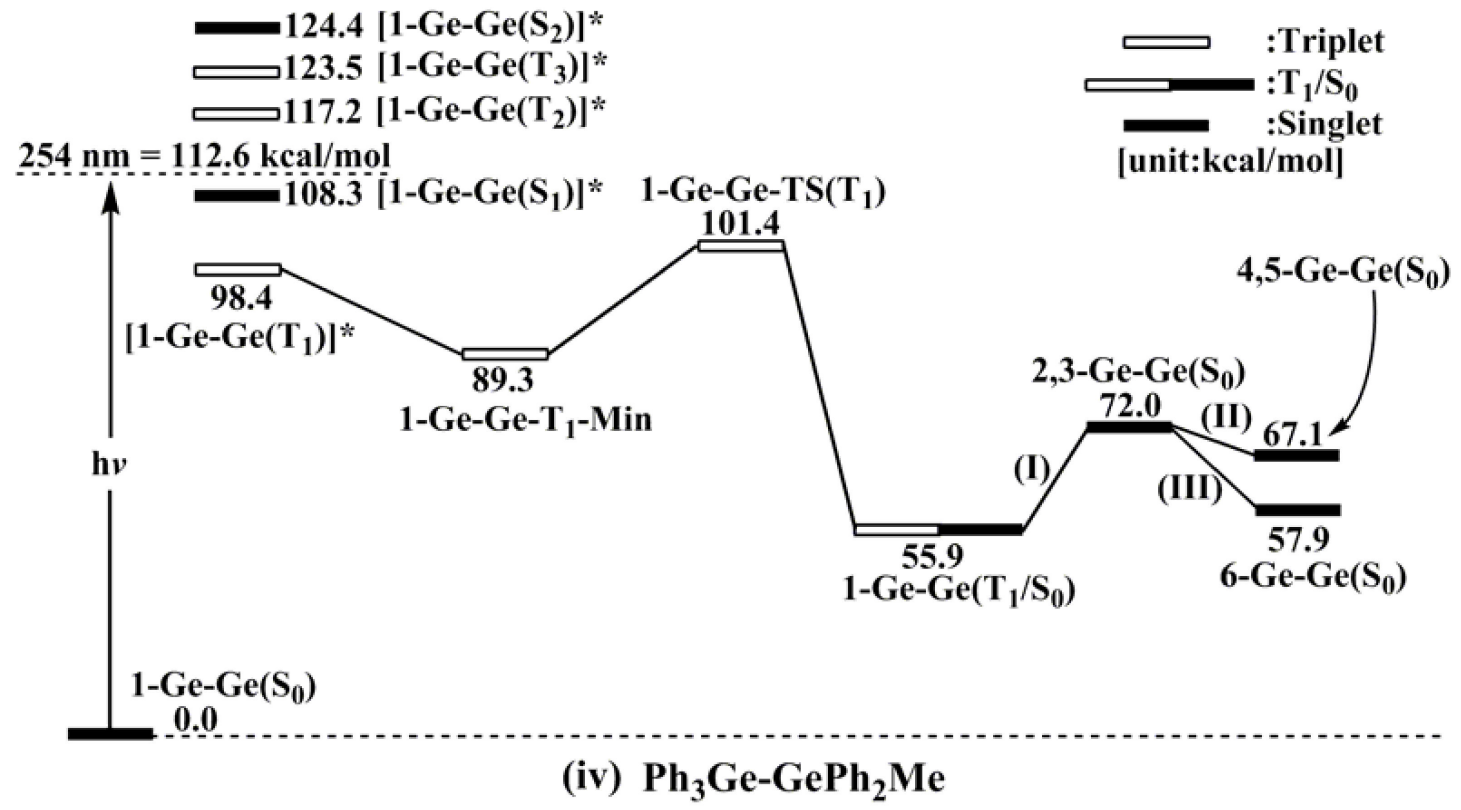
The potential energy profiles for the photochemical isomerization models of Ph_3_M–M′Ph_2_Me (M and M′ = Si and Ge; (**i**–**iv**)). The star represents Frank–Condon. For more information see the text.

**Figure 2:**
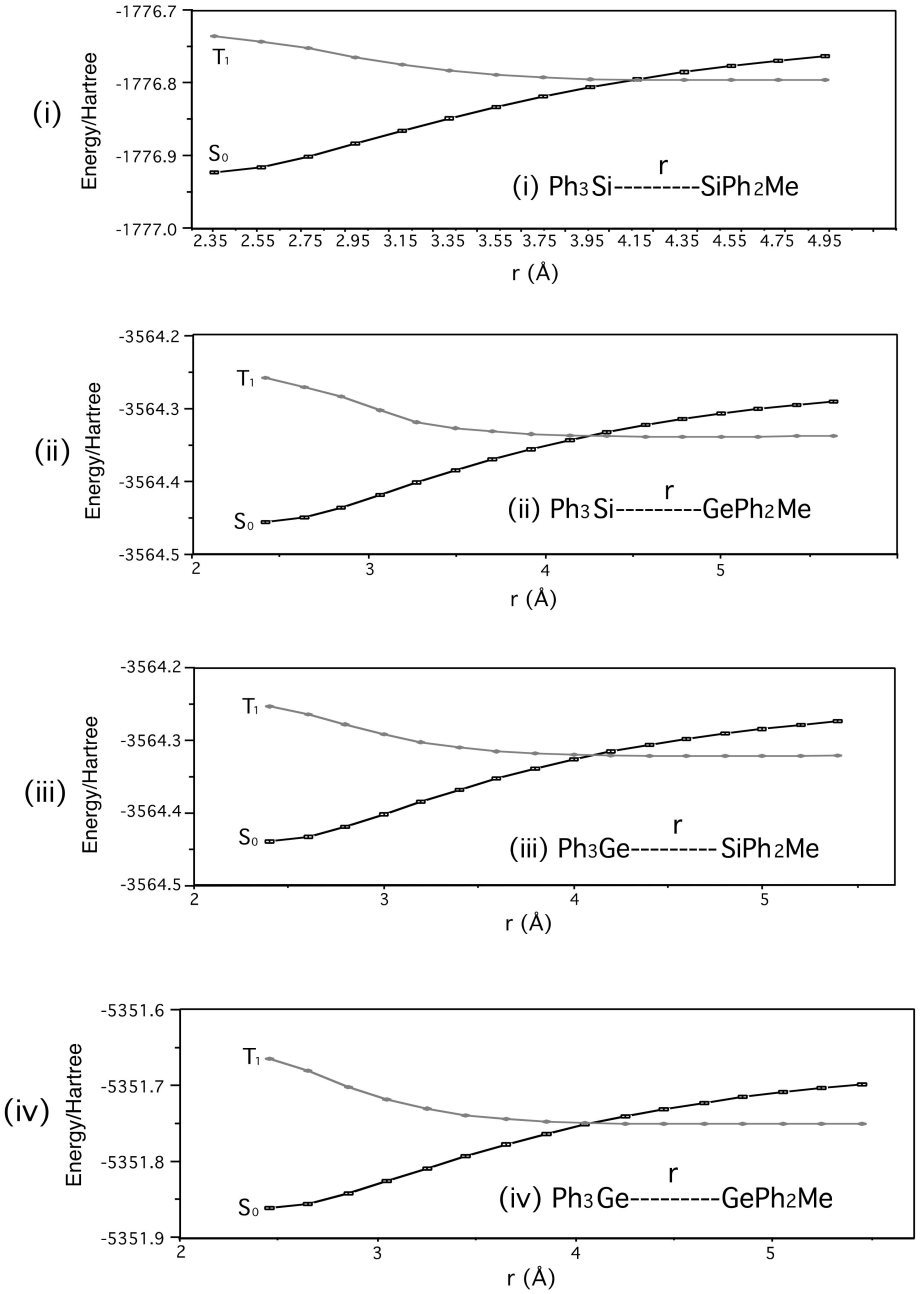
The minimum-energy pathway for a M–M′ bond cleavage in Ph_3_M–M′Ph_2_Me (M and M′ = Si and Ge) along the distance, r, optimized for the S_0_ and T_1_ states at the M06-2X/6-311G(d) level of theory.

**Figure 3:**
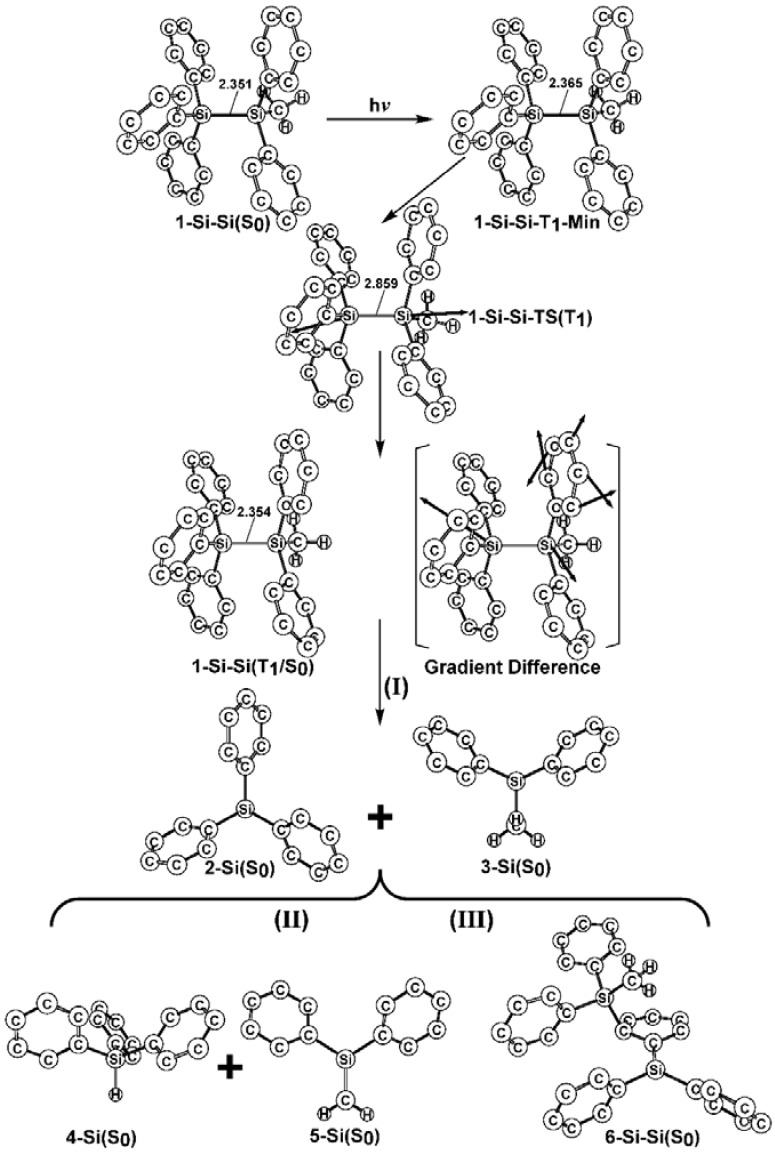
The optimized structures for the photochemical isomerization model of Ph_3_Si–SiPh_2_Me at the M06-2X/6-311G(d) level of theory.

**Figure 4:**
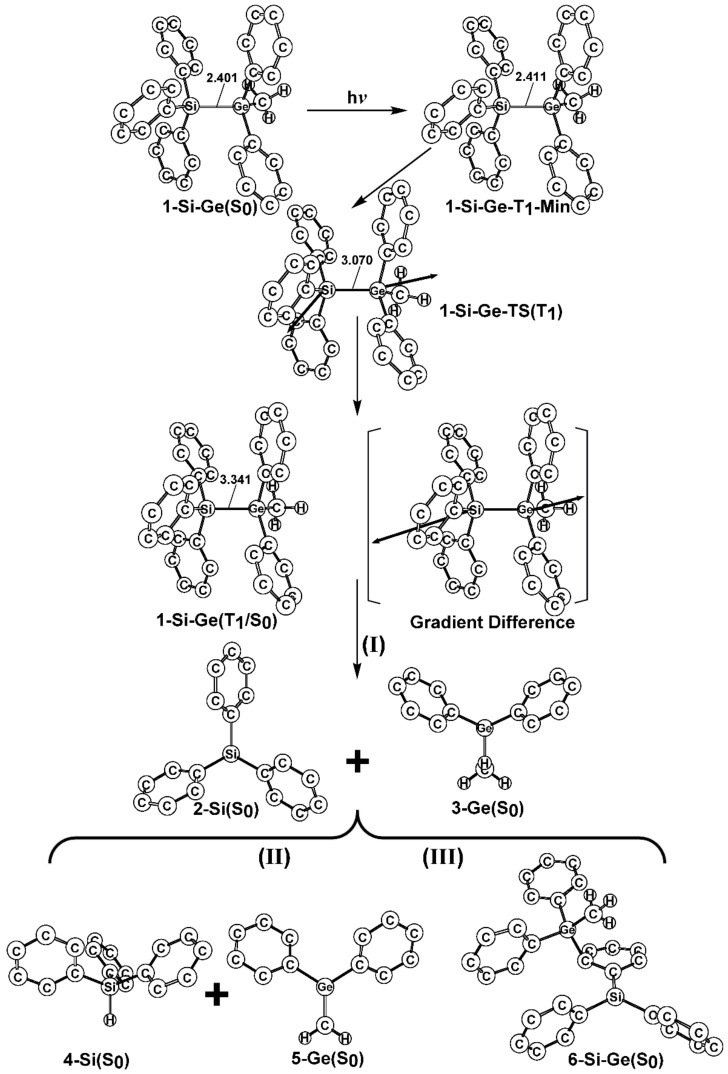
The optimized structures for the photochemical isomerization model of Ph_3_Si–GePh_2_Me at the M06-2X/6-311G(d) level of theory.

**Figure 5:**
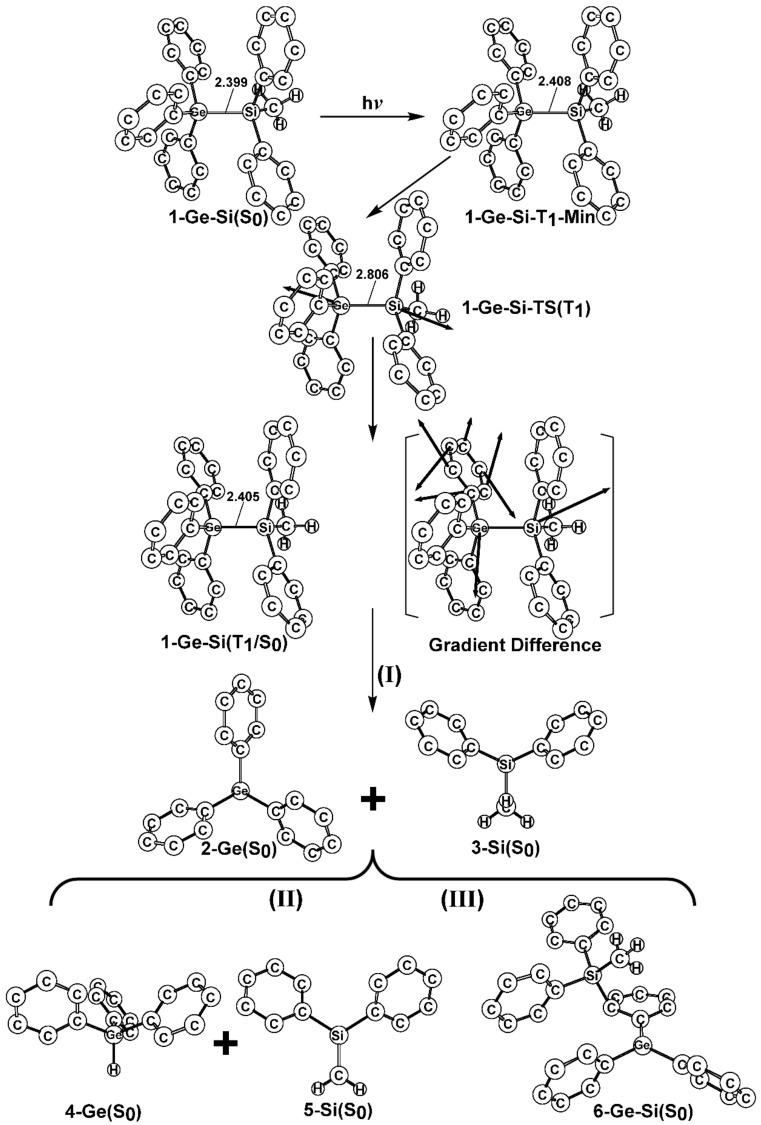
The optimized structures for the photochemical isomerization model of Ph_3_Ge–SiPh_2_Me at the M06-2X/6-311G(d) level of theory.

**Figure 6:**
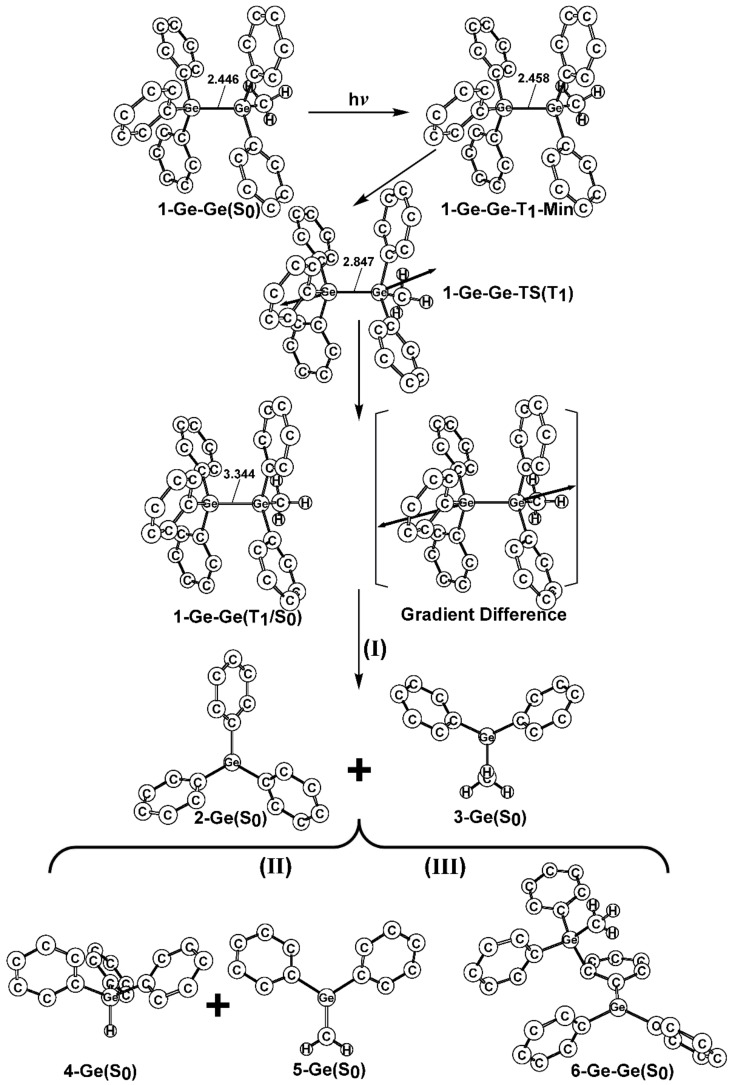
The optimized structures for the photochemical isomerization model of Ph_3_Ge–GePh_2_Me at the M06-2X/6-311G(d) level of theory.
